# Assessment of tantalum nanoparticle-induced MC3T3-E1 proliferation and underlying mechanisms

**DOI:** 10.1007/s10856-021-06606-7

**Published:** 2021-10-23

**Authors:** Chengrong Kang, Yudong Wang, Liang Li, Zhangwei Li, Qianbing Zhou, Xuan Pan

**Affiliations:** Department of Stomatology, The First Affiliated Hospital of Guangdong Pharmaceutical University, Guangzhou, 510080 China

## Abstract

**Objective:**

In our previous study, tantalum nanoparticle (Ta-NPs) was demonstrated to promote osteoblast proliferation via autophagy induction, but the specific mechanism remains unclear. In the present study, we will explore the potential mechanism.

**Methods:**

Ta-NPs was characterized by transmission electron microscopy, scanning electron microscopy, dynamic light scattering, and BET specific surface area test. MC3T3-E1 were treated with 0 or 20 μg/mL Ta-NPs with or without pretreatment with 10 μM LY294002, Triciribine, Rapamycin (PI3K/Akt/mTOR pathway inhibitors) for 1 h respectively. Western blotting was used to detect the expressions of pathway proteins and LC3B. CCK-8 assay was used to assess cell viability. Flow cytometry was used to detect apoptosis and cell cycle.

**Results:**

After pretreatment with LY294002, Triciribine and Rapamycin, the p-Akt/Akt ratio of pathway protein in Triciribine and Rapamycin groups decreased (*P* < 0.05), while the autophagy protein LC3-II/LC3-I in the Rapamycin group was upregulated obviously (*P* < 0.001). In all pretreated groups, apoptosis was increased (LY294002 group was the most obvious), G1 phase cell cycle was arrested (Triciribine and Rapamycin groups were more obvious), and MC3T3-E1 cells were proliferated much more (*P* < 0.01, *P* < 0.001, *P* < 0.05).

**Conclusion:**

Pretreatment with Triciribine or Rapamycin has a greater effect on pathway protein Akt, cell cycle arrest, autophagy protein, and cell proliferation but with inconsistent magnitude, which may be inferred that the Akt/mTOR pathway, as well as its feedback loop, were more likely involved in these processes.

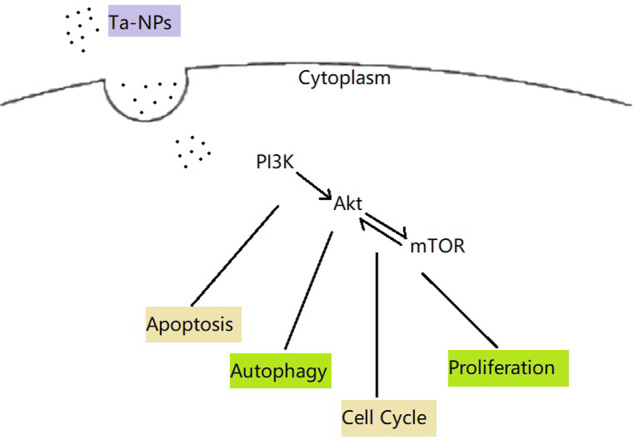

## Introduction

Tantalum has been widely used in medical fields for its excellent biocompatibility, anticorrosive effects, and superior strength. Tantalum-based coatings can promote bone formation, and improve healing and fusion efficacy, especially for patients afflicted by diabetes [1–3]. With the emergence of nanotechnology, nanostructured tantalum, such as tantalum nanoparticles (Ta-NPs), nano-Ta film, and Ta porous scaffolds, have been widely used in dentistry and orthopedics to create an interfacial layer to promote stable cellular adhesion, proliferation, and differentiation [4–7].

Many scholars proposed that tantalum nanotube arrays could enhance cellular adhesion, proliferation, and differentiation [8]. Hierarchical structure features, combined with micron and nanoscale tantalum, were found to have possessed higher surface hydrophilicity and enhanced resistance to corrosion. It facilitated enhanced cell adherence and spreading during initial culture stages (up to 24 h post-initiation) and enhanced cell proliferation, maturation and mineralization within 14 days post-initiation of the culture period [9, 10]. In addition, another in vitro and in vivo study came to similar conclusions that hierarchical micro-nano surfaces significantly increased cellular activities, the bone-to-implant contact area, and newly derived bone volume[11]. These findings could facilitate a fascinating strategy for achieving fast and stable fixation.

Porous tantalum, combine with the dual advantages of hierarchical micro-nano tantalum surfaces and bone trabecular-like porous structures, promoted cell adhesion and proliferation and showed good biocompatibility and osteo-compatibility in vitro as well as in vivo [12, 13]. Moreover, porous tantalum combined with bone marrow stromal stem cells (BMSCs) and bone marrow mesenchymal stem cells (BMMSCs) had even higher efficacies upon osteogenesis [14, 15].

Ta-NPs could promote osteoblast proliferation in low concentrations (12.5 μg/mL), but inhibit proliferation in high concentrations (≥25 μg/mL). This kind of phenomenon was found to be related to autophagy and oxidative stress [16]. In another study, autophagy was proved to be involved in Ta-NPs induced osteoblast proliferation and played a promoting effect [7]. However, this article had no further study on the potential signal pathway.

The PI3K/Akt/mTOR signaling pathway is a classical pathway that has been widely studied. It plays a key role in transcription, translation, metabolism, and regulates a series of cellular processes, including cell proliferation, the cell cycle, autophagy, apoptosis, DNA repair, and so on [17–19]. Therefore, the PI3K/AKT/mTOR signaling pathway has increasingly emerged as a target for disease therapeutic treatments. In many types of cancer treatments, pathway inhibitors and analogs are used to intervene in the PI3K/Akt/mTOR pathway, and thus regulate autophagy and other cellular processes, as well as influence tumor growth [20, 21]. But the drug resistance was the trickiest problem in cancer treatments. Studies have shown that dynamic feedback loops or cross-links between PI3K/Akt/mTOR itself or other related pathways were associated with the drug resistance [22–25].

Based upon these findings, we sought to verify whether the PI3K/Akt/mTOR signaling pathway and its feedback loop participated in Ta-NPs induced autophagy and osteoblast proliferation.

## Materials and methods

### Chemicals and chemical suspensions

Ta-NPs were purchased from Sigma-Aldrich (St Louis, MO, USA; product number 593486). Antibodies against LC3B, (p-)PI3K, (p-)Akt, (p-)mTOR, β-actin, and LY294002 (PI3K inhibitor, LY), Triciribine (Akt inhibitor, API) were all purchased from Cell Signaling Technology (CST, Beverly, MA, USA). Rapamycin (mTOR inhibitor, Rapa) was purchased from Sigma-Aldrich. The Annexin-V-FITC apoptosis detection kit was purchased from Thermo Fisher Scientific (USA). Cell Counting Kit-8 (CCK-8, Dojindo, Japan) assays were used to evaluate measures of cell viability.

Ta-NPs were characterized by transmission electron microscopy (TEM, Mic JEM-1011, JEOL, Japan), scanning electron microscopy (SEM, Nova Nano 430, FEI, Finland), and dynamic light scattering (DLS). Ta-NPs suspension was prepared as described in the previous study [7]. LY, API, and Rapa were dissolved according to the manufacturer’s instructions to form stock solutions which were frozen at −20 °C until further use. Upon use, stock solutions were diluted to working concentrations with α-MEM as needed.

### Cell treatment

MC3T3-E1 mouse osteoblasts (Cell Bank of Shanghai Institute of Life Sciences, Chinese Academy of Sciences, China) were cultured in 6-well plates at a density of 1 × 10^4^ cells/well with 2 mL of α-MEM or were cultured in 96-well plates at a density of 1 × 10^3^ cells/well with 100 μL of α-MEM (for CCK-8 assay only) for 24 h to allow for cell adherence. Next, cells were treated with 0 or 20 μg/mL Ta-NPs for another 24 h with or without 10 μM of LY, of API, or of Rapa pretreatment for 1 h respectively.

### Measures of protein expression via Western blotting

At predetermined time steps, we thrice rinsed cells with PBS and then lysed them with lysis buffer (Whole Cell Lysis Assay, KeyGEN, China) for 30 min on ice. Next, all cell lysates were collected and centrifuged at 10, 000 g for 10 min at 4 °C. We next collected supernatants and then quantified constituents using BCA Protein Quantitation Assays (Beyotime, China). Equivalent amounts of lysate proteins from each sample were loaded onto sodium dodecyl sulfate-polyacrylamide gels and were then electrophoretically transferred to polyvinylidene fluoride membranes (Merck Millipore, Billerica, MA, USA). Transferred membranes were then thrice rinsed (5 min each rinse) with 1 × TBS containing 0.05% Tween-20 (TBST) and were then blocked with 5% nonfat milk in TBST for 30 min at room temperature. Next, membranes were incubated with primary antibodies against p-PI3K, PI3K, p-Akt, Akt, p-mTOR, mTOR, LC3B, and β-actin (1:1000, rabbit antibodies) overnight at 4 °C. We then washed the membranes with TBST, incubated with a horseradish peroxidase-conjugated secondary antibody (1:2000, anti-rabbit antibodies) for 1 h at room temperature, and thrice washed them with TBST. Finally, antibody-bound membranes were detected using ECL Blotting Detection Reagent (Pierce, USA) and the grayscale values for protein bands were analyzed using Image Lab Software (Bio-Rad, USA).

### Cell viability by CCK-8 assay

At predetermined time steps, cell plates were thrice washed with PBS and were then added into 100 μL of fresh α-MEM and 10 μL of CCK-8 reagent. After co-culture for 2 h, the absorbance of the supernatant was quantified at 540 nm using a multi-well plate reader (Multiskan GO, Thermo Scientific, USA). Next, we determined the relative viability of cells using the following formula: relative viability = [(As-Ab)/(Ac-Ab)] × 100%, where As is the experimental optical density (OD), Ac is the control OD, and Ab is the blank OD. At least 5 wells per treatment group and experimental conditions were examined in 3 independently replicated experiments.

### Apoptosis by flow cytometry

At indicated time steps, cell mediums were carefully removed and preserved for later assessment. Cells were thrice rinsed with PBS, were digested with trypsin (without EDTA), and were then collected by centrifugation (1000 rpm, 5 min). Next, cells were re-suspended in PBS and then centrifuged twice (1000 rpm, 5 min) to wash thoroughly. The retained cell mediums described above were added before the final centrifugation. Then, we added 100 μL of pre-cooled binding buffer to suspend the cells, added 5 μL of Annexin V, added PI, and gently mixed samples for 20 min at room temperature without light. Finally, cells were re-suspended in 400 μL of pre-cooled binding buffer and were preserved without light at 4 °C.

### Cell cycle by flow cytometry

At indicated time steps, cells were digested by trypsin and then centrifuged for 5 min (1000 rpm), then were washed twice with PBS and finally collected by centrifugation (1000 rpm, 5 min). Next, we fixed cells with 70% ethanol at 4 °C for 12 h and washed twice with PBS to remove residual ethanol. Then, 100 μL RNase A was added and samples were submerged in a warm bath for 30 min at 37 °C, followed with 30 min of incubation with 400 μL of DNA-binding dye propidium iodide (PI) at 4 °C without light. Finally, we used flow cytometry (BD Biosciences, Franklin Lakes, NJ, USA) to facilitate detections of cell cycle phases based upon measures of DNA content.

### Statistical analyses

Quantitative results were presented as the mean ± standard deviation (SD) and were normalized using one-way analysis of variance (ANOVA). Those data that passed the normality tests were analyzed with the least significant difference test (LSD); those data that did not pass the normality tests were analyzed with a Dunnett’s test. All analyses were conducted using SPSS 22.0 software (SPSS Inc., Chicago, IL, USA). Statistical measures were considered significant when *P* values < 0.05.

## Results

### Characterization of Ta-NPs

**Fig. 1:**
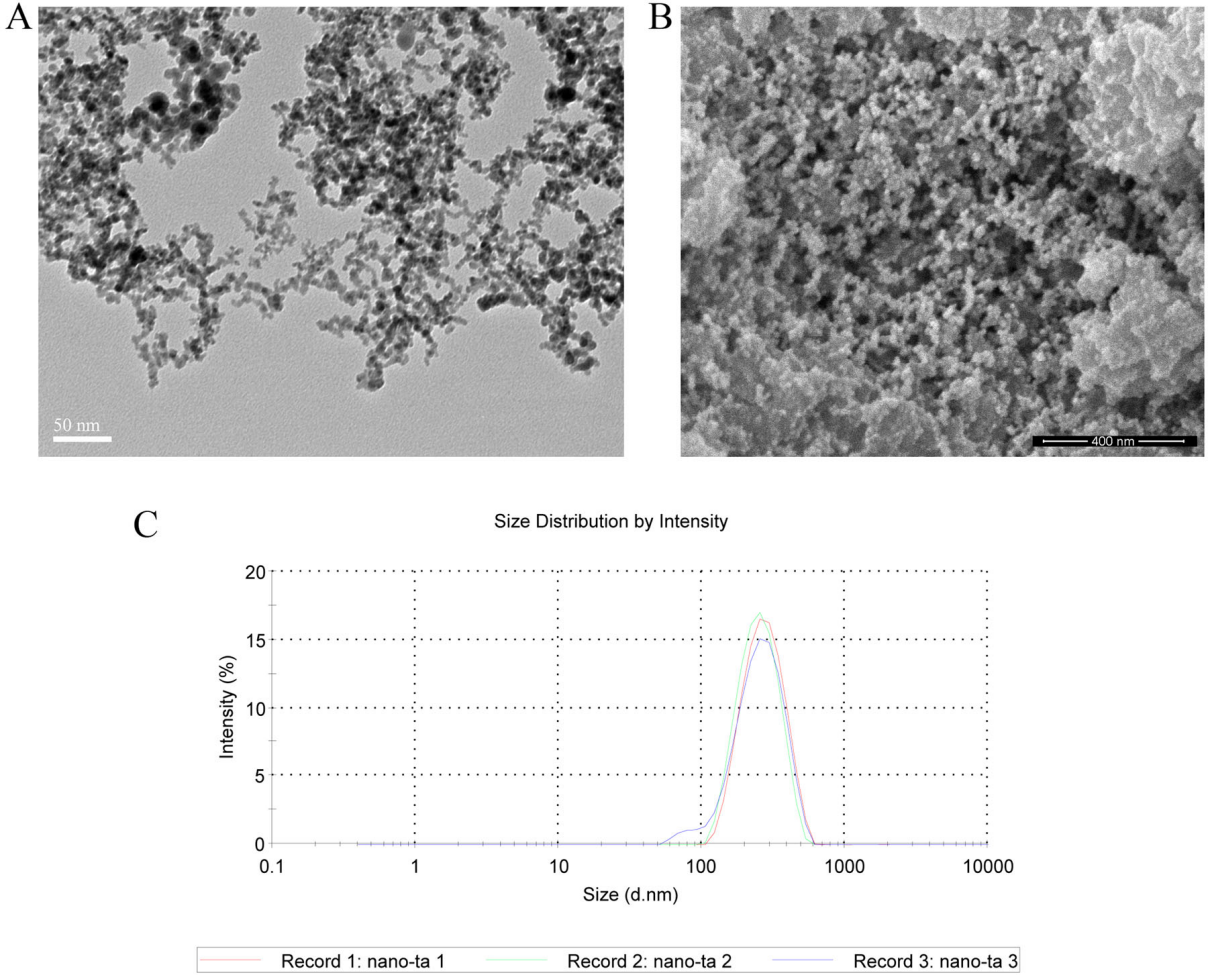
Characterization of Ta-NPs. TEM (**A**) and SEM (**B**) images showed primarily spherical shapes. Magnification: ×200,000. **C** DLS measurement

Ta-NPs were primarily spherical with primary diameters of 8–15 nm and hydrodynamic sizes of 292 nm (Fig. 1) [7]. In addition, the specific surface area for Ta-NPs = 78.19 m^2^/g.

### Protein expression

**Fig. 2:**
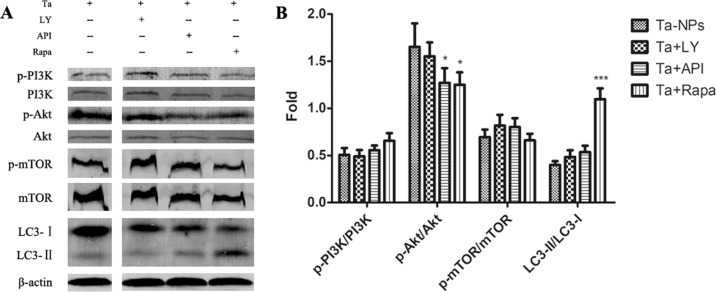
Expression of pathway proteins and autophagy protein (**A**) and quantified in column diagram (**B**). MC3T3-E1 was pretreated with or without 10 μM of LY, API, or Rapa respectively for 1 h followed with 20 μg/mL Ta-NPs treatment for another 24 h. *P < 0.05, ***P < 0.001 vs. Ta-NPs alone treated group

Western blotting was used to detect the expression levels of pathway proteins and autophagy protein LC3B. Compared with Ta-NPs alone treated group, the pathway protein P-Akt /Akt was downregulated in the API and Rapa pretreated groups (*P* < 0.05), and the autophagy protein LC3-II/LC3-I was further upregulated in the Rapa pretreated group (*P* < 0.001) (Fig. 2).

### Cell viability by CCK-8 assay

**Fig. 3:**
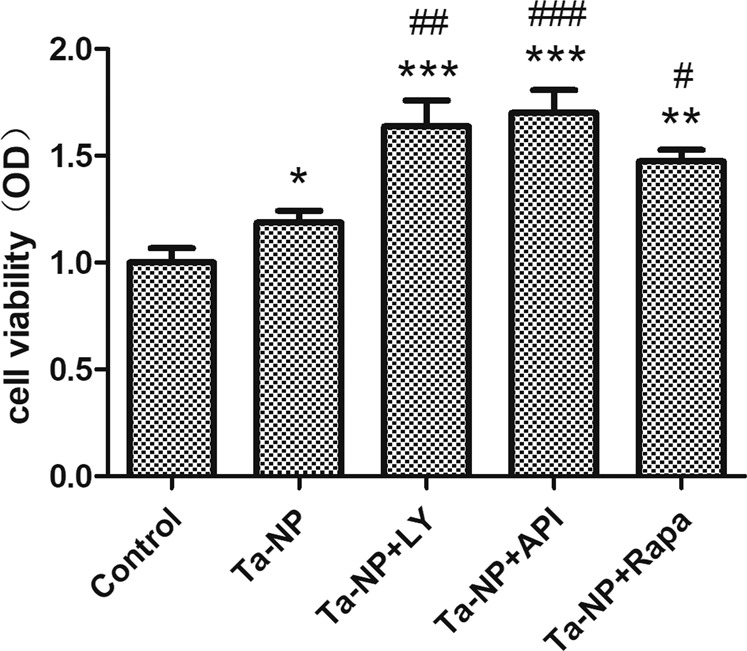
Cell viability. MC3T3-E1 was pretreated with or without 10 μM of LY, API, or Rapa respectively for 1 h followed with 20 μg/mL Ta-NPs treatment for another 24 h. At least 5 wells per condition were examined in 3 independently replicated experiments. *P < 0.05, **P < 0.01, ***P < 0.001 versus control group; ^#^*P* < 0.05, ^##^*P* < 0.01, ^###^*P* < 0.001 vs. Ta-NPs alone treated group

The CCK-8 assay was used to measure cell viability (Fig. 3). Results indicated that Ta-NPs treatment could promote the proliferation of MC3T3-E1. Moreover, the pretreatment of LY, API, or Rapa all significantly further increased the cell viability (*P* < 0.01, *P* < 0.001, *P* < 0.05, respectively), especially the API pretreated group.

### Cell apoptosis by flow cytometry

**Fig. 4:**
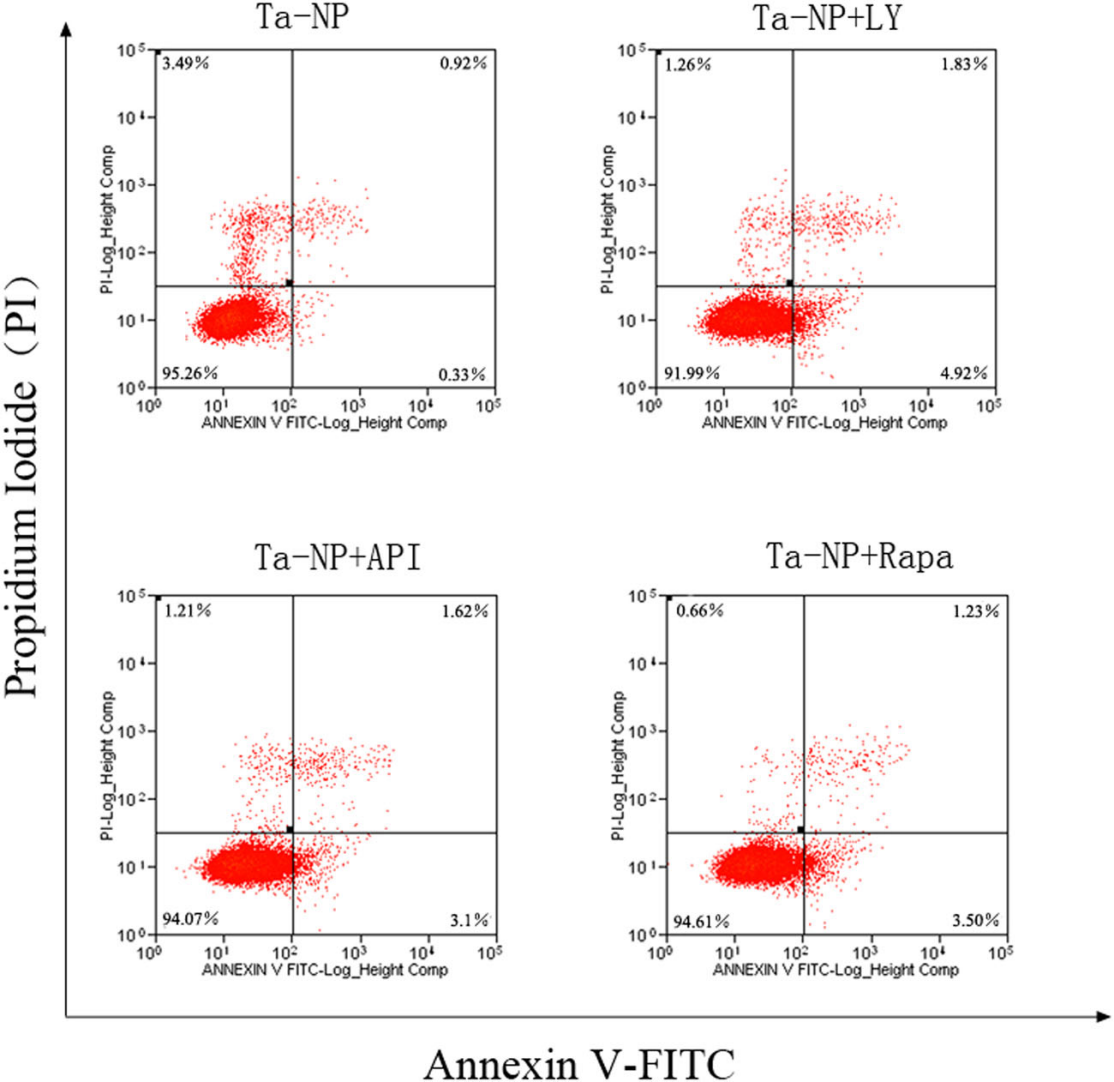
Cell apoptosis. MC3T3-E1 was pretreated with or without 10 μM of LY, API, or Rapa respectively for 1 h followed with 20 μg/mL Ta-NPs treatment for an additional 24 h

Having detected the autophagy and proliferation of MC3T3-E1, we further explored the apoptosis and cell cycle using flow cytometry. Results indicated that the pretreatment of LY, API, and Rapa all upregulated cell apoptosis from a level of 0.33% in the Ta-NPs alone treated group to levels of 4.92%, 3.10%, and 3.50%, respectively (Fig. 4).

### Cell cycle by flow cytometry

**Fig. 5:**
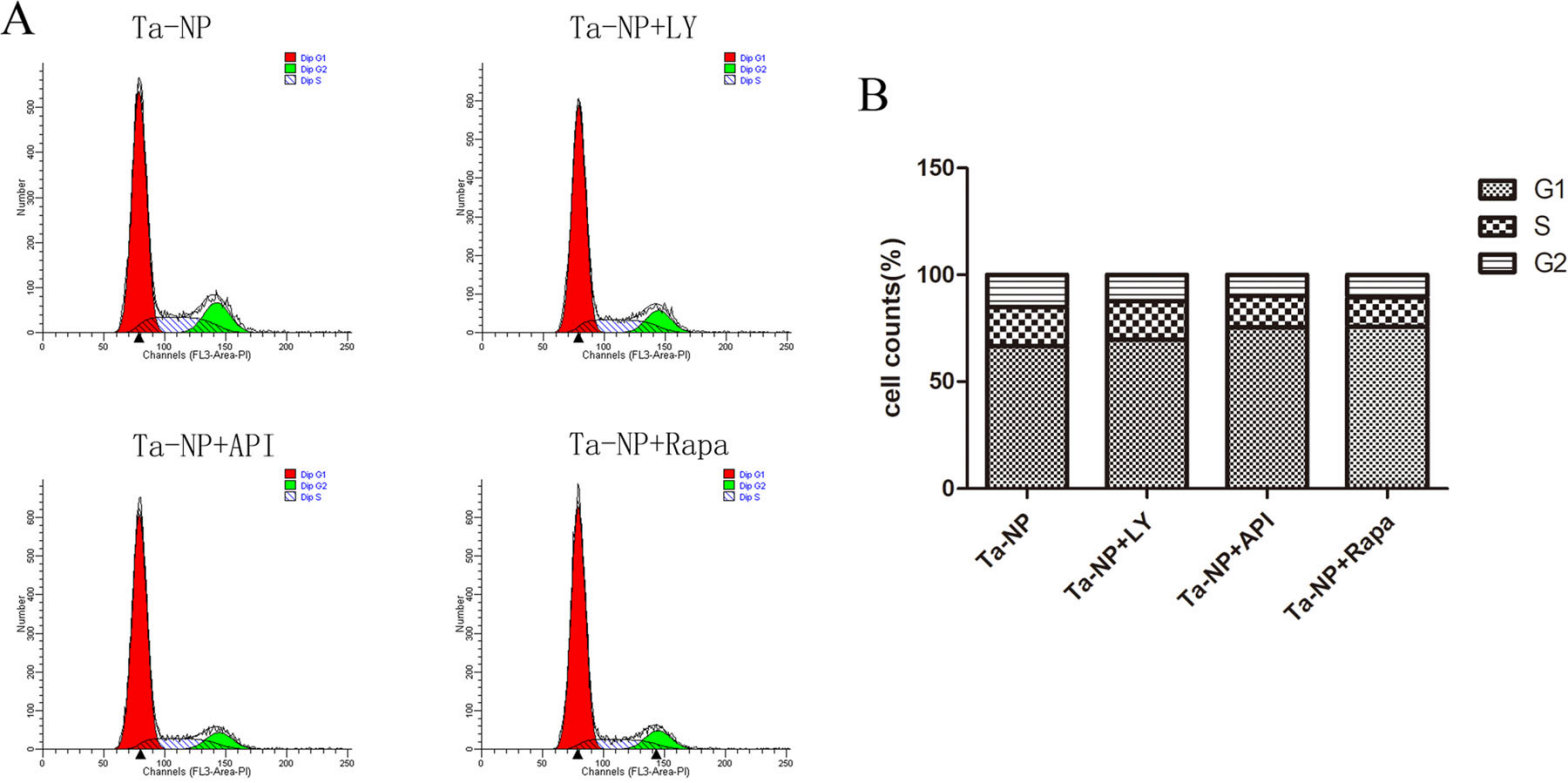
Cell cycle in coordinate diagram (**A**) and quantified in colume diagram (**B**). MC3T3-E1 was pretreated with or without 10 μM of LY, API, or Rapa respectively for 1 h followed with 20 μg/mL Ta-NPs treatment for an additional 24 h

Finally, we assessed the dynamics of cell cycle phases based upon sample DNA content (Fig. 5). Results indicated that the pretreatment of LY, API, and Rapa all increased the proportions of cells in the G1 phase and decreased cells in the S and G2 phases, especially for the API and Rapa pretreated groups.

## Discussion

In our previous research, we proved that autophagy played a positive role in Ta-NPs induced osteoblast proliferation. In this study, we further explore the mechanism involved. The Western blotting findings indicated that only the level of p-Akt/Akt in the API and Rapa pretreated group was lower than that in the Ta-NPs treated group. Meanwhile, the autophagy protein LC3-II/LC3-I in Rapa pretreated group was further upregulated. Did only the Akt participate? And why was the p-Akt/Akt expression in Rapa pretreated group also downregulated? Was there a feedback loop between mTOR and Akt in Ta-NPs treated MC3T3-E1?

The mTOR is a central regulator of cell growth and metabolism and is available in two complex forms: mTORC1 and mTORC2. mTORC1 is sensitive to rapamycin and its function is tightly regulated by PI3K/Akt; mTORC2 was found to have been indirectly inhibited by rapamycin-induced ROS [26, 27]. Moreover, mTORC1 is involved in multiple feedback loops [28–31], including the negative feedback loop between mTORC1 and Akt. Activated mTORC1 could feedback inhibit Akt phosphorylation at Ser473 [31, 32]. mTORC2 is also involved in some feedback loops including a positive feedback loop between mTORC2 and Akt [27, 33, 34]. The positive mTORC2-Akt feedback loop has been revealed as follows: After activation of upstream PI3K, PDK1 activates Akt by way of phosphorylation of Thr308. The partially activated Akt then phosphorylated Sin1 at T86 and enhanced mTORC2 kinase activity, that ultimately led to feedback phosphorylation of Akt Ser 473. Finally, this resulted in the full activation of Akt [35].

**Fig. 6:**
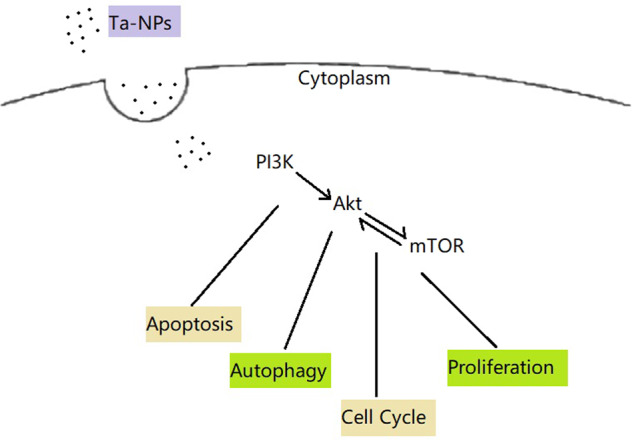
Schematic diagram of the Ta-NPs induced effects on MC3T3-E1. Note: Ta-NPs are endocytosed and activated the mTOR signaling pathway to induce apoptosis, regulate autophagy, cell cycle, and cell proliferation. Ta-NPs Tantalum nanoparticles

Rapamycin is the popular mTOR inhibitor and autophagy inducer. Rapamycin could simultaneously act on mTORC1 and mTORC2 and playing completely different roles according to the action time and concentration. Acute inhibition (for 1 h) increased insulin signaling and glucose uptake, while chronic inhibition (for 24–48 h) induced insulin resistance and impaired insulin-mediated glucose uptake [22, 36]. Similarly, different concentrations of rapamycin resulted in a biphasic effect. Rapamycin in low concentration (10 nM) increased the Akt and ERK phosphorylation through a mTORC1-dependent negative feedback loop, high dose of rapamycin (1000 nM) inhibited the Akt and ERK phosphorylation via the mTORC2 mediated positive feedback loop [37]. In the present study, the action time and concentration of rapamycin were 1 h and 10 μM. Thus, rapamycin may act more on mTORC2 and the positive feedback loop between mTORC2 and Akt may play a leading role. This may explain to some extent that the p-Akt/Akt level reduced only in API and Rapa pretreated group and the LC3-II/LC3-I upregulated in Rapa pretreated group. Published research has indicated that mTORC2 was tightly associated with the development of the cell cycle through an Akt-dependent manner [37]. As shown in the present study, high doses of rapamycin inhibited Akt phosphorylation and caused cell cycle arrest in the G1 phase, especially in the API and Rapa pretreated group. The two-way feedback loop in that rapamycin leads to Akt and ERK activation at low concentrations, whereas lead to Akt and ERK inhibition at high concentrations may probably offer an anti-cancer therapy strategy. Therefore, these findings implied that the Akt/mTOR signaling pathway and its feedback loop participated in the cell regulation of Ta-NPs treated MC3T3-E1 (Fig. 6).

In conclusion, pretreatment with LY, API, Rapa upregulated the autophagy and apoptosis, led to cell cycle arrest, but the cell proliferation was still maintained. That’s a little confusing. In addition to the protective effect of autophagy on cell survival, there maybe exist some other proliferative mechanisms that need to be further studied and discovered.

## Conclusion

The Akt/mTOR signaling pathway as well as its feedback loop was involved in Ta-NPs induced autophagy and proliferation. However, further research is needed to elucidate the exact signal molecule involved in the Akt/mTOR feedback loop.
